# A Novel *DES* L115F Mutation Identified by Whole Exome Sequencing is Associated with Inherited Cardiac Conduction Disease

**DOI:** 10.3390/ijms20246227

**Published:** 2019-12-10

**Authors:** Lung-An Hsu, Yu-Shien Ko, Yung-Hsin Yeh, Chi-Jen Chang, Yi-Hsin Chan, Chi-Tai Kuo, Hsin-Yi Tsai, Gwo-Jyh Chang

**Affiliations:** 1 Cardiovascular Division, Department of Internal Medicine, Chang Gung Memorial Hospital, Chang Gung University College of Medicine, Tao-Yuan 33305, Taiwan; c12037@adm.cgmh.org.tw (Y.-S.K.); yys0tw@yahoo.ca (Y.-H.Y.); chijenformosa@gmail.com (C.-J.C.); s851047@hotmail.com (Y.-H.C.); chitai@adm.cgmh.org.tw (C.-T.K.); sunny66house@yahoo.com.tw (H.-Y.T.); 2 Graduate Institute of Clinical Medical Sciences, Chang Gung University, Tao-Yuan 33305, Taiwan; gjchang@mail.cgu.edu.tw

**Keywords:** cardiac conduction disease, exome sequencing, desmin

## Abstract

Inherited cardiac conduction disease (CCD) is rare; it is caused by a large number of mutations in genes encoding cardiac ion channels and cytoskeletal proteins. Recently, whole-exome sequencing has been successfully used to identify causal mutations for rare monogenic Mendelian diseases. We used trio-based whole-exome sequencing to study a Chinese family with multiple family members affected by CCD, and identified a heterozygous missense mutation (c.343C>T, p.Leu115Phe) in the desmin *(DES)* gene as the most likely candidate causal mutation for the development of CCD in this family. The mutation is novel and is predicted to affect the conformation of the coiled-coil rod domain of DES according to structural model prediction. Its pathogenicity in desmin protein aggregation was further confirmed by expressing the mutation, both in a cellular model and a CRISPR/CAS9 knock-in mouse model. In conclusion, our results suggest that whole-exome sequencing is a feasible approach to identify candidate genes underlying inherited conduction diseases.

## 1. Introduction

The functional components of the cardiac conduction framework include impulse-generating nodes and the impulse-propagating His-Purkinje system. In cardiac conduction disease (CCD), the integrity of the conduction framework is flawed, such that impulse generation, impulse propagation, or both will be slowed or even blocked, and life-threatening rhythm disturbances may follow. CCD can be caused by an acquired injury such as ischemia or drug toxicity, related to heart diseases such as cardiomyopathy or congenital defects, associated with neuromuscular diseases, or rarely, an isolated finding [1,2]. The familial clustering of idiopathic CCD has led to the identification of many mutations in genes encoding ion channels (Voltage-Gated Sodium Channel Subunit Alpha (*SCN5A*), Hyperpolarization Activated Cyclic Nucleotide-Gated Potassium Channel 4 (*HCN4*), Potassium Inwardly-Rectifying Channel Subfamily J Member 2 (*KCNJ2*)), cardiac transcription factors (Homeobox protein Nkx-2.5 (*NKX2-5)*, T-box transcription factor (*TBX5)*, gap junctions (Connexin 40 (*Cx40*)), energy metabolism regulators (5’-AMP-activated protein kinase subunit gamma-2 (*PRKAG2*)) and structural proteins (lamin A/C (*LMNA*)) that cause progressive conduction system disorder without structural heart disease [1,2]. Inherited forms of CCD are uncommon; however, each new mutation provides an insight into the molecular mechanisms controlling the development and function of the cardiac conduction framework [3].

Clinically, genetic testing can help to identify patients at risk of CCD before it manifests. However, genetic testing techniques, knowledge about the candidate genes, and the size of the pedigrees may hinder efforts to identify relevant genetic defects. This can restrict the analysis to priori-identified candidate genes, and may fail to reveal causal genes in familial CCD if no mutations are found among the known candidate genes. In addition, the application of linkage analysis may be jeopardized by the small size of the pedigrees. In this regard, next generation whole genome or exome sequencing may be helpful in overcoming such limitations [4]. In this study, we investigated a Chinese family with inherited CCD that manifested as sinus arrest, atrial fibrillation, and atrioventricular conduction disturbance. Using trio-based, whole-exome sequencing techniques, we found a novel point mutation located in *DES* encoding the muscle-specific, type-III, intermediate filament (IF), desmin. Structural modeling predicted that this mutation may have an effect on *DES* function. Both cellular and CRISPR/CAS9 knock-in mice modeling further supported its pathogenicity in desmin protein aggregation.

## 2. Results

### 2.1. Trio-based Exome Sequencing and Variant Filtration and Prioritization

**Figure 1:**
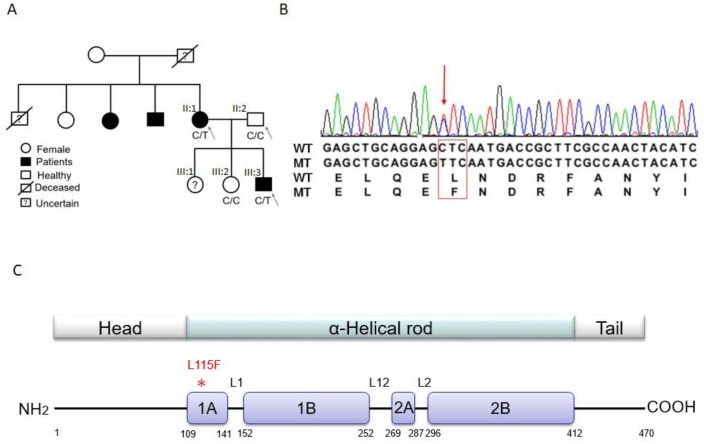
Mutation identification. (**A**) Pedigree of the studied family. The pedigree shows the affection statuses, individual identifiers, and genotypes at *DES* c.C343T. The phenotype of sample III:1 was uncertain. The samples (marked by arrows) II:1, II:2, and III:3 (proband) were exome sequenced. (**B**) Sequencing result showing the heterozygous *DES* c.C343T (p.L115F) mutation. WT, wild-type allele; MT, mutant allele. (**C**) Schematic of desmin protein. * L115F mutation in the 1A region.

The first set of analyses was performed on a Chinese family with inherited CCD (Family K; the pedigree is shown in Figure 1A). CCD presented with early onset, symptomatic arrhythmia, including a long pause after atrial fibrillation, and a combination of sino-atrial and atrioventricular nodal conduction blocks, which contributed to pacemaker implantation (Table 1). As shown in Figure 1A, the study family members consisted of one affected son (proband, III:3), one affected mother (II:1), one unaffected father (II:2), and one unaffected daughter (III:2). The phenotype of an elder sister (III:1) of the proband was uncertain, since she refused to participate in the study. One maternal uncle and one maternal aunt of the proband were also affected and had had permanent pacemaker implantations at other hospitals; however, they were unavailable for DNA testing. None of the affected family members showed muscular dystrophy or other organ system involvement, and none had evidence of cardiomyopathy or LV dysfunction (Table 1). There was also no history of sudden cardiac death (SCD) in the family members. Trio-based exome sequencing was performed on the proband and his parents. On average, 90.73% of the exome was covered at least 20-fold. Overall, we identified around 115,819 single nucleotide variants (SNVs) and 7007 insertions/deletions (InDels) from three study samples (39,229 to 41,896 variants per subject). Our selection strategy in this trio-based sequencing was to find a rare and functional variant that matched an assumed autosomal dominant inheritance model. In an autosomal dominant hereditary disease, both the proband and one of the parents have symptoms. Heterozygous mutations that exist in both the proband and the symptomatic parent, but are absent from the healthy parent, are potential causal mutations. The number of variants left after each step of the candidate variant finding pipeline are shown in Table 2. The pipeline filtered out most of the synonymous SNVs that were considered to be neutral variants, and nonsynonymous SNVs that were reported in the dbSNP142 and 5000 Exomes (minor allele frequencies (MAF) > 0.01) databases. More than 200 candidate variants were left and entered into function prediction programs. Fifty-six candidate variants matched the autosomal dominant model and remained after passing through the pipeline (Table 2). Among the 56 candidate genes, only *ACAD9, AGL, DES, FAH,* and *PLEC* genes have been linked to heart diseases in the Online Mendelian Inheritance in Man (OMIM) and PubMed databases. The goal of this study was to identify a novel and family-specific mutation. Of these five remaining SNVs, we thus filtered out four variants that had been reported in the Genome Aggregation Database (gnomAD) (MAF ≤ 0.00011) and were considered to be known rare variants. Consequently, only the *DES* c.343C>T, p.Leu115Phe (p.L115F) variant was absent from the gnomAD database, and remained the most likely causative variant of CCD.

### 2.2. Validation of Candidate Genes

We conducted direct sequencing validation for the *DES* p.L115F variant in the affected mother and son, the healthy father and sister (Figure 1A,B), and 100 unrelated healthy controls, which showed that the *DES* p.L115F variant was a novel and family-specific mutation. Further, the *DES* p.L115F variant was also absent from the Taiwan BioBank database, which contains genome sequences of 1517 healthy Taiwanese people (https://taiwanview.twbiobank.org.tw/index).

### 2.3. DES Wild-Type/Mutation Structure Modeling

**Figure 2:**
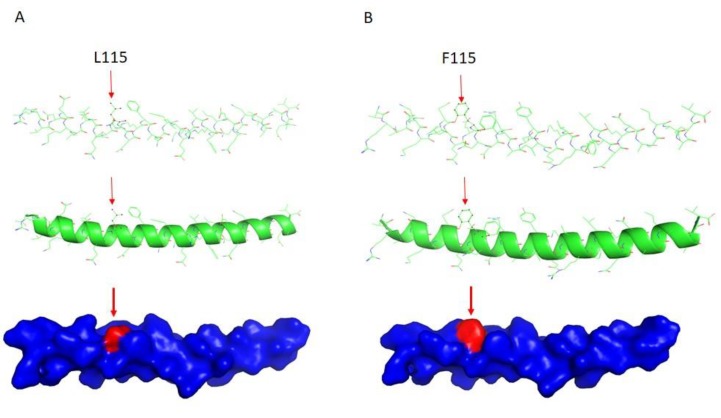
Structural models of DES. (**A**) Model of the wild-type (WT) DES (residues 109−141) was built using the Coot program (https://www2.mrc-lmb.cam.ac.uk/personal/pemsley/coot/). (**B**) The L115F DES mutant model. L115 and F115 are marked with arrows.

IF structures are characterized as a central alpha-helical, coiled-coil rod domain consisting of four alpha-helical segments (Figure 1A,B and Figure 2A,B) bridged by three linker regions [5]. Of note, the *DES* mutation p.L115F sits at the 1A region (Figure 1C), which has been suggested to be involved in the elongation of filaments and dimer formation [6,7]. We thus used the model building program Coot [8] to build a structural model containing this site. As shown in Figure 2, replacement with phenylalanine at this site led to a conformational change, possibly due to the benzyl side chain of phenylalanine 115. This may have caused the mutant form to assemble as a dimer more loosely or assemble into a dimer in a different way, which may than have hindered the subsequent formation of the coiled-coil rod domain, or altered the biophysical properties of the mutated protein, leading to increased susceptibility to stress-induced filament breakdown.

### 2.4. Cytoplasmic Aggregate Formation Caused by the Desmin Mutant in Transfected Cells

**Figure 3:**
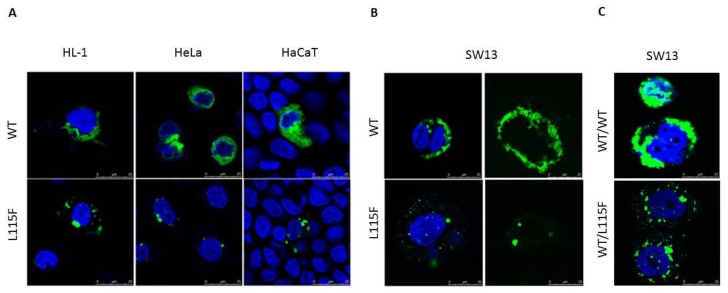
Expression of desmin in cultured cells. (**A**) HL-1 atrial cardiomyocytes, HeLa, and HaCaT cells transfected with wild-type (WT) or p.L115F desmin in fluorescent expression vectors, showing cytoplasmic aggregates with p.L115F independent of the transfected cell type. (**B**) SW-13 cells transfected with WT or p.L115F desmin in fluorescent expression vectors, showing p.L115F desmin unable to develop an extended filamentous network as WT. (**C**) SW-13 cells cotransfected with the same amount of WT and p.L115F desmin expression vectors, showing that WT/p.L115F desmin was unable to develop an extended filamentous network as WT/WT. Scale bar = 25 µm.

Expression constructs of desmin wild-type (WT) and p.L115F fused at the C-terminus to GFP were generated and transiently transfected in HL-1, HeLa, and HaCaT cells. In agreement with other studies [9,10,11,12,13], we detected cytoplasmic aggregates with p.L115F, independent of the transfected cell type (Figure 3A). To test the ability of p.L115F desmin to assemble into a de novo filament network, WT and mutant desmin expression vectors were transiently transfected into IF-free SW-13 human adrenocortical carcinoma cells (Figure 3B). In contrast to the WT, p.L115F desmin was unable to develop an extended filamentous network as featured by protein aggresome formation throughout the cytoplasm. Because of the heterozygosity of the patients, expression analyses were also performed on SW-13 cells cotransfected with equal amounts of WT and p.L115F desmin expression vectors. As shown in Figure 3C, the SW-13 cells cotransfected with WT, and the mutant also failed to develop an extended filamentous network as with the homozygous WT. These findings suggested that *DES* p.L115F had a dominant negative effect on filament assembly.

### 2.5. Immunohistochemistry and Electron Microscopy in des ^L114F^ Knock-in Mice

**Figure 4:**
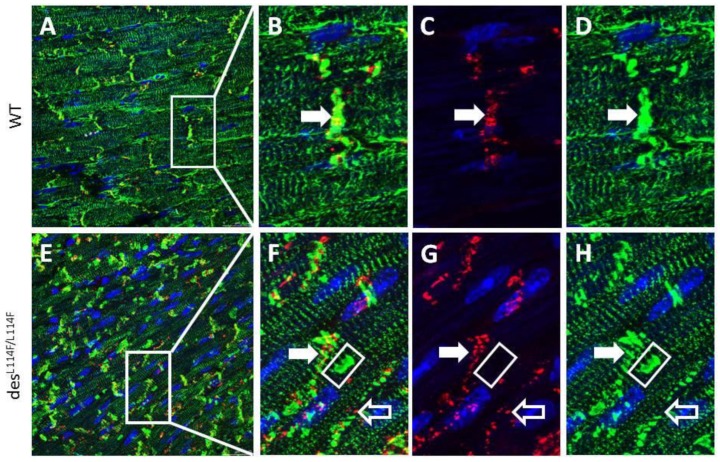
Confocal microscopy of ventricular myocardium from wild-type (WT) (**A**) and *d**es*****^L114F/L114F^** mice (**E**). (**B**–**D**) Zoomed region of the white square in (A). (**F**–**H**) Zoomed region of the white square in (**E**). Representative confocal images showed numerous desmin-positive but connexin 43-negative (marked by white square in Figure **F**–**H**) irregularly amorphous aggregates among the myofibrils, and altered connexin 43 distribution (open arrow) in the des^L114F/L114F^ mice. The arrows indicate colocalization of desmin and connexin 43 at intercalated discs. Green signals indicate desmin; red signals indicate connexin 43 gap junctions; yellow signals indicate the colocalization of desmin and connexin 43; and blue signals indicate nuclei stained with 4′,6-diamidino-2-phenylindole (DAPI). Scale bar = 25 µm.

**Figure 5:**
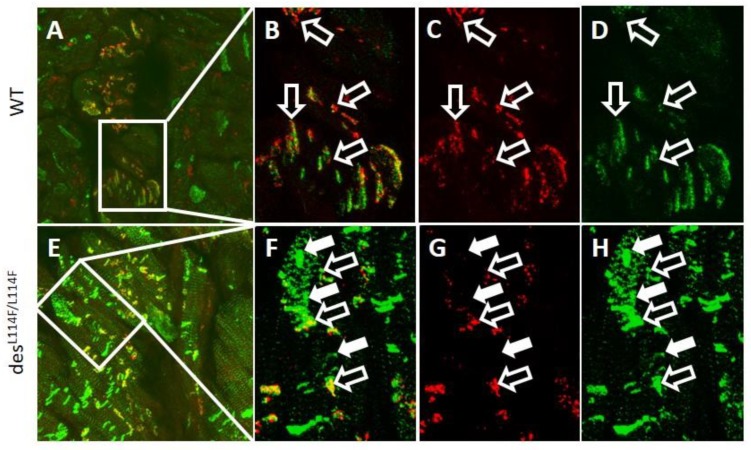
Confocal microscopy of ventricular Purkinje fibers from wild-type (**A**) and *des*^L114F/L114F^ mice (**E**). (**B**–**D**) Zoomed region of the white square in (**A**). (**F**–**H**) Zoomed region of the white square in (**E**). Representative confocal images showed numerous desmin-positive but connexin 40-negative (arrows in Figure **F**–**H**) irregularly amorphous aggregates in the Purkinje fibers from *des*^L114F/L114F^ mice, but not from wild-type mice. Anti-connexin 40 immunostaining aided in identifying cardiac conduction cells (Purkinje fibers). The open arrows indicate colocalization of connexin 40 and desmin at intercalated disc. Green signals indicate desmin; red signals indicate connexin 40 gap junctions; and yellow signals indicate the colocalization of desmin and connexin 40. Scale bar = 25 µm.

**Figure 6:**
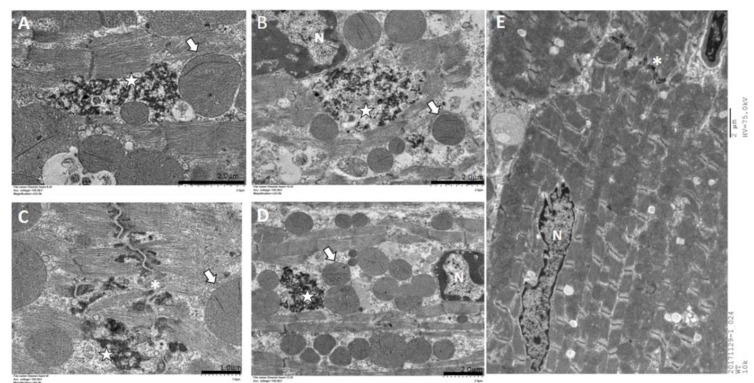
Transmission electron microscopy. **A**–**D**, 7-week-old *des*^L114F/L114F^ homozygote; **E**, 12-week-old wild type; Electron microscopy of ventricular myocardium from 7-week-old *des* ^L114F/L114F^ homozygote revealed numerous highly electron-dense, amorphous, irregularly lobulated aggregates (marked by stars) in the intermyofibrillar space (**A**) and enriched at the levels of peri-nucleus (**B**) and intercalated discs (**C**), associated with disintegrated and thinning of myofibrils (**B**,**D**), as well as aggregates of swollen and disorganized mitochondria (arrows). N, nucleus; *, intercalated disc. (**A**,**B**,**D**,**E**) scale bar = 2 µm; (**C**) scale bar = 1 µm.

**Figure 7:**
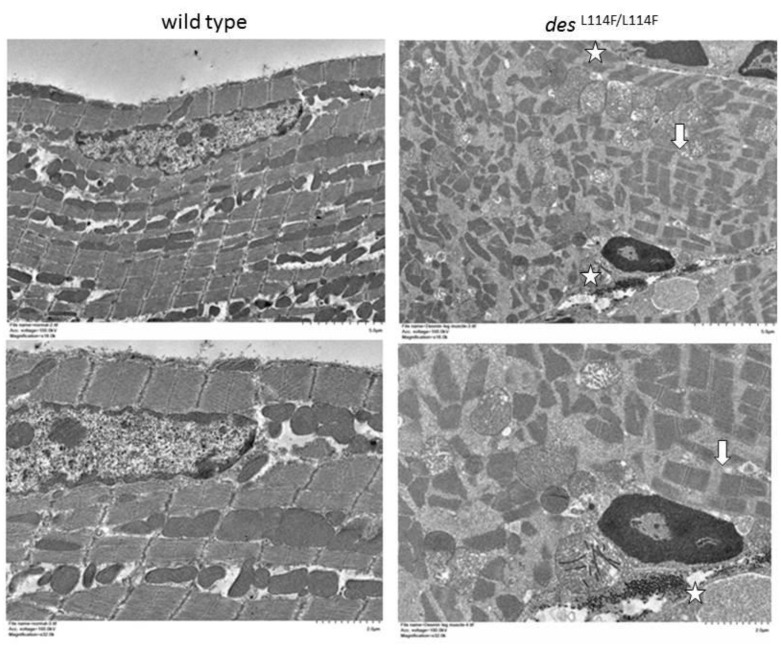
Transmission electron microscopy of the soleus muscle from wild-type (WT) and *des*^L114F/L114F^ mice. Electron microscopy of the soleus muscle from 7-week-old *des*^L114F/L114F^ mice revealed numerous electron-dense aggregates (marked by stars) in the perinuclear region with sarcomeres disrupted and discontinued at Z-discs (arrows). Upper panel, scale bar = 5 µm; lower panel, scale bar = 2 µm.

We further generated a *des*^WT/L114F^ knock-in (KI) founder mouse using a CRISPR/CAS9 system, which harbored the ortholog of the human missense mutation L115F. *Des*^WT/^^L114F^ mice were viable and born at an expected Mendelian ratio. However, only a few *des*^L114F^^/^^L114F^ mice were obtained in litters from *des*^WT/^^L114F^ intercrosses at weaning, and they rarely survived to 3 weeks, indicating that the *des* Leu114Phe homozygous mutant was perinatal lethal. The surface electrocardiogram (ECG) study of 3-month-old heterozygous *des*^WT/^^L114F^ mice displayed no significant differences in PR, QRS, and QT intervals, compared to the WT mice. However, homozygous *des*^L114F^^/^^L114F^ mice were too fragile to obtain good surface ECG signals for analysis. Similar to the results of desmin knockout mice models [14,15], only homozygous *des*^L114F^^/L114F^ mice showed an earlier onset myopathy phenotype. Ventricular myocardium was available from mice of different genotypes. Confirming the in vitro results of the cell culture experiments with the mutant *DES* L115F, we found a high density of desmin aggregates within the ventricular myocardium of *des*^L114F^^/ L114F^ mice in both confocal and electron microscopy (EM) studies, which was undetectable in the ventricles of WT mice and barely detectable in the ventricles of *des*^WT/L114F^ KI mice. Confocal images of immunoreactive desmin signals revealed notable changes in the distribution of *des* ^L114F/L114F^ mice. As shown in Figure 4, ventricular myocytes from WT mice displayed a regular, sarcomeric arrangement of desmin staining, as well as discrete, high intensity desmin signals at intercalated discs, indicating interactions between desmin and cell–cell adhesion junctions. In contrast, ventricular myocytes in *des*^L114F/L114F^ mice displayed large perinuclear clusters of desmin signals. The normal sarcomeric arrangement and, in particular, the cell–cell junction localization of desmin were disrupted in *des* ^L114F/L114F^ ventricular myocytes. Thus, the expression of mutant desmin seemed to disturb the normal distribution of desmin filaments in ventricular myocytes and electrical junctions. We further evaluated the distribution of the major gap junction channel protein, connexin 43 (Cx43). Compared to the WT, an increased distribution of Cx43 in the lateral border of cells was observed in the ventricular myocardium of *des*^L114F/L114F^ (Figure 4G). Because of conduction block in the patients, double immunostaining with anti-Cx40 and anti-desmin was performed to investigate whether the desmin mutant affected the His-Purkinje system. As shown in Figure 5, the accumulation of desmin aggregates was also demonstrated in the Purkinje fibers of *des*^L114F^^/L114F^ mice, but not in the Purkinje fibers of WT mice. Similarly, EM of *des*^L114F/L114F^ myocardium revealed highly electron-dense, amorphous, and irregularly lobulated aggregates in the intermyofibrillar space, enriched at the levels of peri-nucleus and intercalated discs (Figure 6). The disintegration and thinning of the myofibrils were also noted. Moreover, mitochondrial abnormalities, including the loss of proper morphology, swelling, and the loss of cristae structure were detected in *des*^L114F/L114F^ mice, which are compatible with a common pathological feature among desmin-related myopathies (Figure 6). The homozygous *des*^L114F/L114F^ KI mouse also developed a prominent desmin aggregate pathology in skeletal muscle. As shown in Figure 7, EM of the soleus muscle from *des*^L114F/L114F^ mice revealed electron-dense aggregates in the perinuclear region and among the myofibrils. Furthermore, almost all the structures of each sarcomere were disrupted and discontinued at Z-discs.

## 3. Discussion

We used trio-based whole exome sequencing to evaluate a family with autosomal dominant CCD and identified a heterozygous missense mutation (NM_001927.3, c.343C>T, p.L115F) in the *DES* gene as the most likely causative variant. This mutation was predicted to cause damage by in silico analysis, and to affect conformation of the desmin coiled-coil rod domain by Coot software. Subsequently, we confirmed that the mutation showed complete cosegregation with the disease phenotype in the family and the absence of the mutation in an additional 100 healthy controls. This mutation is novel and does not appear in public databases such as ClinVar, the Exome Aggregated Consortium, gnomAD, or Taiwan BioBank database. An in vitro study of transient cell transfections in different cell lines, including HL-1 atrial cardiomyocytes, and the HeLa, HaCaT, and SW-13 cell lines showed that the novel *DES* p.L115F mutation could form cytoplasmic aggregates. We further generated *des* L114F KI mice using a CRISPR/CAS9 system, which harbored the ortholog of the human missense mutation L115F. The homozygous *des*^L114F/L114F^ KI mouse also developed prominent desmin aggregate pathology in cardiac tissue and skeletal muscle. Our results allowed us to classify *DES* p.L115F as being pathogenic according to the American College of Medical Genetics and Genomics guidelines on the interpretation of genetic variants [16].

Dysfunction of the desmin network owing to mutations in the desmin gene or post-translational modifications leads to cardiac and skeletal myopathies, jointly called desmin-related myopathies or desminopathies. A phenotype-genotype correlation meta-analysis study showed that mutations in the rod 2B domain of desmin are mainly found in patients with both skeletal and cardiac muscle phenotypes, whereas head and tail domain mutations are predominant in patients with an isolated cardiac phenotype [17,18]. However, the clinical phenotypes are very variable, even within the same family. The majority of desmin mutations result in cardiac manifestations, including conduction system defects and all forms of cardiomyopathy, with dilated cardiomyopathy (DCM) being the most frequent, followed by restrictive (RCM), hypertrophic (HCM), arrhythmogenic right ventricular dysplasia/cardiomyopathy (ARVD/C) and their combinations [19,20]. The major phenotype in our study family was closer to isolated CCD and manifested as extensive atrial disease, tachy-brady arrhythmias, and atrioventricular conduction block. The identified mutation p.L115F is located in segment 1A of the rod domain of desmin. The mutation belongs to the consensus sequence ‘LNDR’, which is absolutely conserved in IF proteins among eukaryotes [6,7]. The amino acid L115 is a “d position” amino acid of the heptade, which appears to be responsible for the hydrophobic seam connecting desmin alpha-helices within the filament’s supercoil. Substitution of leucine by phenylalanine, which has a benzyl side chain within the hydrophobic core, may affect the stability of the coiled coil, and thus, the oligomerization equilibria. Two other mutations have been identified on both sides of L115. The mutation E114del was recently identified in a Uruguayan family affected by myopathy and severe cardiomyopathy [21]. Members of this family displayed arrhythmias, conduction block, and SCD. An EM study of skeletal muscle biopsies showed deposits of granulofilamentous material at the subsarcolemmal and intermyofibrillar levels, as well as megamitochondria at the subsarcolemmal level [16]. In addition, the mutation p.N116S was identified as a de novo mutation in a patient with ARVC and terminal heart failure [9]. Immunohistochemical investigations of explanted hearts have demonstrated the accumulation of desmin immunoreactive aggresomes in the RV and left ventricle [9].

In cardiac muscles, desmin is enriched at intercalated discs, and provides a major component of Purkinje fibers [22]. Thus, atrioventricular conduction abnormalities requiring the implantation of a permanent pacemaker are a frequent feature of desminopathy [17]. The immunohistochemistry and EM of cardiac muscles from the homozygous *des*^L114F/L114F^ KI mice showed desmin aggregates and granulofilamentous electron-dense materials, typical features of desminopathy. Given that the mutation was identified in a family with conduction defects, we also showed desmin aggregates deposited along the Purkinje fibers of *des* ^L114F/L114F^ KI mice. This finding of desmin aggregates supports the hypothesis that mutant misfolded desmin protein serves as a seed for the development of protein inclusions in desminopathy. The aggregates could interrupt the continuity and overall organization of the desmin network throughout the cell and lead to increased mechanical vulnerability of myofibrils and Purkinje fibers. Notably, the highly abnormal desmin-staining pattern shown in *des*^L114F/L114F^ myocardium was enriched at the levels of peri-nuclei and intercalated discs, and was associated with an increased distribution of Cx43 to the lateral border of cells. A previous report demonstrated that the remodeling of gap junctions and altered distribution of Cx43 led to slowing of ventricular conduction in a transgenic mouse model mimicking 7-amino acid deletion (R172-E178) in humans [23]. Furthermore, defective linkage between desmosomes and the mutant desmin may have impaired the formation and stabilization of electrical junctions in the heart, which may have led to conduction abnormalities and increased arrhythmogenesis [24]. In contrast to a dominant negative effect of the L115F mutation shown in the cellular model and the heterozygous genotype observed in the patients, only homozygous *des*^L114F^^/L114F^ mice showed an earlier onset myopathy phenotype in the L114F KI mice model. This could be because the process of damage to cardiac conduction with the L115F heterozygous mutation is age-dependent, and takes time to develop. Accordingly, the heterozygous mice were not observed for long enough for late manifestations of the cardiac phenotype to develop, and further, long-term study is warranted. Recently, Clemen et al. generated R349P desmin KI mice which harbored the ortholog of the human desmin missense mutation R350P [25]. These mice developed an age-dependent desmin-positive protein aggregation pathology, skeletal muscle weakness, dilated cardiomyopathy, as well as cardiac conduction defects and arrhythmias. Desmin-positive protein aggregates were present in both heterozygous and homozygous mice, but clear manifestations of progressive skeletal muscle weakness and morphological signs of myopathic changes were only present in the latter, which is similar to our KI mice. Their findings suggest that the total or focal disruption of the extrasarcomeric IF cytoskeleton, instead of the presence of desmin protein aggregates per se, is the major factor that triggers progressive muscle fiber damage [25]. 

There are several limitations to this study. First, we could not determine whether the *des^L114F^* KI mice recapitulated the CCD phenotype observed in the study family with the use of non-invasive ECG and Holter monitoring or invasive electrophysiology studies. Second, the pathogenic mechanism underlying the association between the *DES* mutations and CCD remains unclear. Further studies are needed to investigate whether L115F can cause further changes in the subcellular localization and turnover of direct desmin-binding partners, subsequent cell apoptosis in the cardiac conduction system, or the loss of desmin-dependent gene regulatory function, as previously reported in some of *DES* missense mutations [11,12,25,26]. 

## 4. Materials and Methods

### 4.1. Ethics Statement

The study protocols were approved by the Human Research Ethics Committee at Chang Gung Memorial Hospital (Chang Gung Medical Foundation Institutional Review Board 98-0615B, 2 April 2009; 101-1088A3, 19 June 2012; and 103-5044B, 8 October 2014) and were conducted in accordance with the Declaration of Helsinki Principles. Written informed consent was obtained from each subject. All animal experimental procedures were approved by the Institutional Animal Care and Use Committee of Chang Gung University (Taoyuan, Taiwan; IACUC No. CGU16-056, 20 September 2016), and the experiments were performed in accordance with the guidelines. 

### 4.2. Study Population

Genomic DNA was prepared from the venous blood of a variety of affected and unaffected individuals following standard procedures. The first set of analyses was performed on a Chinese family with inherited CCD (Family K; pedigree in Figure 1A). The samples II:1, II:2, and III:3 (proband) were exome sequenced. The validation study included the sample III:2 and a cohort of 100 additional unrelated controls recruited from a population receiving routine health examinations.

### 4.3. Exome Sequencing and Variant Calling

Trio-based whole-exome sequencing (WES) was performed to investigate SNVs or small InDels in three family members (affected mother, affected son and healthy father). High-quality genomic DNA (100 ng) was enriched in Ion AmpliSeq Exome RDY plates using Ion AmpliSeq HiFi Mix (Ion Torrent, Carlsbad, CA, USA). The resulting 240–280 bp amplicons were handled with FuPa Reagent (Ion Torrent) to partially digest the primers and phosphorylate the amplicons, which were then ligated to Proton adapters and purified according to the manufacturer’s instructions (Ion Torrent). Libraries were quantified by quantitative PCR and then sequenced on an Ion Proton platform. The raw sequencing output data of the Ion sequencer were processed using the Torrent sequence generation algorithm, and then subjected to standard bioinformatics analysis. First, TMAP (https://github.com/nh13/TMAP) was used to align reads to the reference sequence. The alignment information was stored in Binary Alignment and Mapping (BAM) format files. SNVs and InDels were all called using the Torrent Variant Caller (https://github.com/iontorrent/Torrent-Variant-Caller-stable) (TVC) and annotated using Ion Reporter software (IR). Quality control was performed at each stage of the analysis pipeline for the raw data, the alignment, and the called variant. The data were aligned and mapped to the NCBI reference genome (GRCH37/h19), and were further analyzed and filtered using IR software (Ion Torrent, Carlsbad, CA, USA). Following alignment to the human reference genome, the candidate variants finding workflow was approached as follows: (1) select exonic or splicing site variants, (2) select heterozygous variants, (3) exclude synonymous SNVs, (4) exclude the variants with minor allele frequencies >1% in the dbSNP (version 142) or in the 5,000 Exomes Project, (5) exclude benign variants (SIFT < 0.05 or Polyphen2 > 0.85), (6) select variants coexisting within the affected mother and affected son, (7) exclude the variants coexisting within the affected mother, affected son and unaffected father, and (8) select variants within hereditary cardiovascular disease-related genes.

### 4.4. Mutation Validation

The candidate variant was reconfirmed by Sanger sequencing with the new PCR product for the affected mother and son, as well as the healthy father and daughter. Oligonucleotide primers were generated to amplify fragments of genomic DNA containing the identified variant (Table 3). The novel variant that was validated in the affected members was then examined in more than 100 unrelated healthy persons with normal phenotypes to exclude the possibility of polymorphisms. Genotyping was performed by PCR and restriction enzyme digestion, as described in Table 3.

### 4.5. DES Wild-Type/Mutation Structure Modeling

The structural models of the WT desmin and its p.L115F mutant (residues 109–141) were built using Coot [8]. 

### 4.6. Cell Culture 

HeLa (ATCC, CCL-2), HaCaT (kindly provided by Dr Chung), and SW-13 (lack a vimentin IF) (ATCC, CCL-105) cells were cultured in Dulbecco’s modified Eagle medium (DMEM) (GibcoBRL) supplemented with 10% fetal bovine serum (FBS) and 1% penicillin-streptomycin (GibcoBRL). The HL-1 cells (kindly provided by Dr Claycomb) were cultured in Claycomb medium (Sigma-Aldrich, St. Louis) supplemented with 10% FBS, 2 mmol/L L-glutamine, 100 nmol/L norepinephrine, and penicillin/streptomycin. 

### 4.7. Expression Analysis

To characterize the consequences of the *DES* mutation at the cellular level, transient cell transfections were performed in different cell lines with WT or mutated *DES* mRNA expressed as fusions to the C-terminus of green fluorescent protein (pCMV6-AC-GFP, RG205685 for Desmin, OriGene Technologies). Mutations were introduced via site-directed mutagenesis (Q5^®^Site-directed mutagenesis kit, New England Biolabs, Ipswich, MA, USA) with the forward primer 5′-GGAGCTGCAGGAG**T**TCAATGACCGCTTC-3′ and the reverse primer 5′GAAGCGGTCATTGA**A**CTCCTGCAGCTCC-3′. All of the inserts were systematically verified by sequencing. The transfection was performed by incubating 2 μg of fusion protein construct using LipofectAMINE 2000 (Invitrogen, Carlsbad, CA, USA) according to the manufacturer’s instructions. Cells were grown for 24 h. Cell images were captured using a Leica confocal laser-scanning microscope (TCS SP2, Leica, Wetzlar, Germany).

### 4.8. Generation of des^L114F^ Knock-in Mice Using CRISPR/CAS9

**Figure 8:**
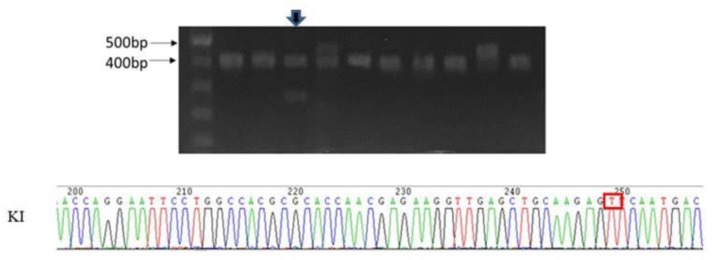
Founder (lane marked by the arrow) containing the knock-in (KI) allele was confirmed by sub-cloning and direct sequencing analysis.

According to the conserved sequence between humans and mice in this region of the desmin gene, the mice L114F mutation corresponds to the human L115F mutation. To introduce a single nucleotide substitution (C to T) within exon 1 of the *des* genomic fragment corresponding to the position of human L115F mutation, four single-guide RNAs (sgRNA) were designed for murine *des* target locus, and their efficiencies were tested in cell lines. One of them (5′-CACGCGCACCAACGAGAAGGTGG-3′) was chosen for the subsequent construction of RNA-guided endonuclease (RGEN) expression vector, which was used for the preparation of DNA templates for in vitro transcription of sgRNA. A T7 polymerase initiation site was added to the 5-prime end of the sgRNA. In order to prevent re-cut of the KI allele by RGEN and to create an *EcoRI* restriction site (underlined), the Mouse-*des*-ssDNA Donor encompassing the sgRNA recognition site (in *italics*) (5′-TGAACCAGGA**A**TTCCTGGC*CACGCGCACCAACGAGAAGGT**T**G*AGCTGCAAGAG*T*TCAATGACCGCTTCGCCAACTACATCGAGAAGGT-3′) was modified using Quickchange (Agilent Technologies) to contain alternate codons that still maintained the translated protein sequence. Four-week-old C57BL/six female mice were super-ovulated and mated with C57BL/6 males. Day 0.5 single cell embryos were isolated and given pronuclear micro-injections using standard methods [27]. The embryos were co-injected with sgRNA at 50 ng/µl, recombinant Cas9 protein at 100 ng/µl, and the Mouse-*des*-ssDNA Donor at 30 ng/µl in DNase/RNase-free micro-injection buffer, 1 mM Tris, 0.25 mM EDTA pH 7.4. Twenty to twenty-five injected embryos were transferred into the oviducts of day 0.5 pseudopregnant CByB6F1 recipient female mice. Genomic DNA was prepared from tail samples of founder mice and their offspring. To establish donor insertion, the mice were genotyped using PCR primers (F: 5′-GGCTCCTCGAGTTCAATGACA-3′, R: 5′-GCCTCTGCAGGTCGTCTATCA-3′), followed by *EcoRI* restriction. The resulting fragments were 436 bp for the WT, and 270 and 166 bp for KI. The PCR products were then directly sequenced or cloned and sequenced (Figure 8). 

### 4.9. Immunohistochemistry 

Adult hearts from the mice of different genotypes were frozen in liquid nitrogen. Mouse heart tissues were fixed overnight at 4 °C in PBS-buffered 4% (wt/vol) paraformaldehyde and embedded in paraffin. Immunohistochemical analyses were performed by confocal microscopy using desmin, connexin 43, and connexin 40 primary antibodies (Abcam, Cambridge, MA, USA) followed by fluorescein isothiocyanate (FITC) or Cy3-conjugated secondary antibodies (Chemicon, Temecula, CA, USA). Nuclei were visualized by DAPI-staining. 

### 4.10. Transmission Electron Microscopy

Adult hearts and soleus muscles from the mice of different genotypes were fixed in 3% glutaraldehyde and 2% paraformaldehyde in 0.1 M cacodylate buffer (pH 7.4) at 4 °C. The specimens were then postfixed in 1% osmium tetroxide (pH 7.4), dehydrated in a graded series of ethanol, and embedded in EPON-812. Thin sections (80 nm) were cut, stained with uranyl acetate and lead citrate, and examined on a Hitachi HT7800 Transmission Electron Microscope (Tokyo, Japan).

## 5. Conclusions

We used trio-based whole exome sequencing to evaluate a family with autosomal dominant CCD, and identified a missense mutation in the *DES* gene as the most likely candidate mutation. Our results indicate that whole exome sequencing is a feasible approach by which to identify candidate genes underlying inherited conduction diseases.

## Figures and Tables

**Table 1:** Demographic and phenotype information of the study family.

	Sex	Conduction Defect	Paf	Age at Echo	LV EF, %	LA, mm	LV, mm	Age at PPM
II:1	F	SND, 3^rd^-degree AVB	yes	55	73	47	46	55
II:2	M	No	No	65	80	37	50	
III:2	F	No	No					
III:3	M	SND, 1^st^-degree AVB	yes	35	64	38	43	35

Paf, paroxysmal atrial fibrillation; SND, sinus node dysfunction; AVB, atrioventricular block; PPM, permanent pacemaker implantation.

**Table 2:** Analysis steps of candidate variants finding workflow in the proband (III:3).

	Steps	Residual SNVs & InDels	Percentage (%)
	TVC filtered variants	39,229	100
1	exonic or splicing site variants	15,206	38.76
2	selected heterozygous variants	8935	22.78
3	excluded synonymous SNVs	4279	10.91
4	excluded the variants with minor allele frequencies >1% in the dbSNP (version 142) or in the 5000Exomes Project	230	0.59
5	excluded benign variants (SIFT < 0.05 or Polyphen2 > 0.85)	226	0.58
6	variants coexisting within the affected mother and affected son	68	0.17
7	excluded the variants coexisting within the affected mother, affected son, and unaffected father	56	0.14
8	variants within hereditary cardiovascular disease-related genes	5	0.01

SNVs, single nucleotide variants; InDels, insertions/deletions; TVC, Torrent Variant Caller.

**Table 3:** Primer sequences and restriction enzyme (RE) used in the analysis of the novel variant.

Gene Name	Primer Sequence	PCR Size (bp)	RE	Allele	Amino Acid Change
DES	(F)-5’-GTCCCGCGTGTACCAGGTGTC-3’, (R)-5’-CCAAGAAAACTCCTGTGCAAGATG-3’	646	SacI	C>T	L115F

RE: restriction enzyme.
